# Identifying novel antimicrobial peptides from venom gland of spider *Pardosa astrigera* by deep multi-task learning

**DOI:** 10.3389/fmicb.2022.971503

**Published:** 2022-08-24

**Authors:** Byungjo Lee, Min Kyoung Shin, Jung Sun Yoo, Wonhee Jang, Jung-Suk Sung

**Affiliations:** ^1^Department of Life Science, Dongguk University-Seoul, Goyang-si, South Korea; ^2^Animal Resources Division, National Institute of Biological Resources, Incheon, South Korea

**Keywords:** antimicrobial peptide, deep learning, multi-task learning, species-specific prediction, spider venom gland transcriptome

## Abstract

Antimicrobial peptides (AMPs) show promises as valuable compounds for developing therapeutic agents to control the worldwide health threat posed by the increasing prevalence of antibiotic-resistant bacteria. Animal venom can be a useful source for screening AMPs due to its various bioactive components. Here, the deep learning model was developed to predict species-specific antimicrobial activity. To overcome the data deficiency, a multi-task learning method was implemented, achieving *F*1 scores of 0.818, 0.696, 0.814, 0.787, and 0.719 for *Bacillus subtilis*, *Escherichia coli*, *Pseudomonas aeruginosa*, *Staphylococcus aureus*, and *Staphylococcus epidermidis*, respectively. Peptides PA-Full and PA-Win were identified from the model using different inputs of full and partial sequences, broadening the application of transcriptome data of the spider *Pardosa astrigera*. Two peptides exhibited strong antimicrobial activity against all five strains along with cytocompatibility. Our approach enables excavating AMPs with high potency, which can be expanded into the fields of biology to address data insufficiency.

## Introduction

The overuse and the misuse of antibiotics have contributed to the increased selective pressure for antibiotic resistance (Cantón and Morosini, 2011). As a result, the emergence and spread of antibiotic-resistant bacterial strains have risen dramatically during recent years and caused at least 35,000 deaths in the United States, according to a 2019 report (Centres for Disease Control and Prevention, 2019). As the widespread of antibiotic-resistant bacteria has become a serious medical threat, there is an urgent need for discovering novel antimicrobial reagents that can directly contribute to the development of next-generation antibiotics. Among various bioactive molecules, peptides are advantageous for therapeutic applications because of their distinctive characteristics and physiological roles, such as immunomodulators, hormones, ligands, and signals (Cunha et al., 2008; Posner and Laporte, 2010; Krumm and Grisshammer, 2015). The intrinsic diversity in the sequence of peptides leads to a broad spectrum of molecular targets with high specificity and selectivity (Tsomaia, 2015). In addition, peptides have low cytotoxicity, low tissue accumulation, and cost-effectiveness compared with small molecules and recombinant proteins (Recio et al., 2017).

Antimicrobial peptides (AMPs) play a key role in the innate immune system, contributing to the defense against pathogens like bacteria, fungi, parasites, and viruses and exhibiting immunomodulatory activities (Yeung et al., 2011; Van Dijk et al., 2016; Mookherjee et al., 2020). Generally, AMPs are less than 50 amino acids (AAs) in length with hydrophobic residues and high positive net charges, leading to interactions with pathogen membranes and intracellular molecules (Huan et al., 2020). By virtue of their non-specific and rapid killing mechanisms against pathogens, AMPs are a promising novel class of antibiotics for preventing and combating antibiotic-resistant bacteria (Nuti et al., 2017; Lei et al., 2019). AMPs are found in almost every organism and are highly abundant in animal venoms. For example, in the antimicrobial peptide database 3 (APD3), 153 AMPs originated from the venom of various species, including spider (Wang et al., 2016). Spiders are among the most successfully evolved taxa of venomous animals, which led to the possession of venom with remarkably diverse mixtures comprising thousands of different components (Langenegger et al., 2019). Therefore, spider venom could be a useful source of novel AMPs (Budnik et al., 2004; Wang and Wang, 2016; Wadhwani et al., 2021).

The increased utilization of deep learning in biological and medical research enabled high-throughput analyses, aided by exponentially accumulating biological datasets (Schmidt and Hildebrandt, 2021). Various deep learning models have been developed for the prediction of miRNA targets, the identification of biological signals, the inference of gene expressions, and the prediction of DNA functions based on large amounts of genomic data (Chen et al., 2016; Lee et al., 2016; Quang and Xie, 2016; Tan et al., 2017). Although deep learning techniques have also been applied in neurotoxic, antibacterial, antifungal, and anticancer peptides discovery, the expansion of similar approaches to some fields is challenging due to the data limitations (Fjell et al., 2009; Lata et al., 2010; Waghu et al., 2016; Meher et al., 2017; Ashfaq et al., 2021; Charoenkwan et al., 2021; Lee et al., 2021). The diversity of bacterial genomes led to distinctive bioactivities of individual strains, as well as numerous defense mechanisms toward antibacterial agents (Zhou et al., 2015). Even though it is not our intention to diminish the importance of developing narrow-spectrum antibiotics, each type of pre-existing method may predict AMPs working on only one or a few types of bacterial species. Thus, we tried to build a generalized model that can predict AMPs working on multiple species to suggest reliable candidates of novel antibiotics.

**Figure 1:**
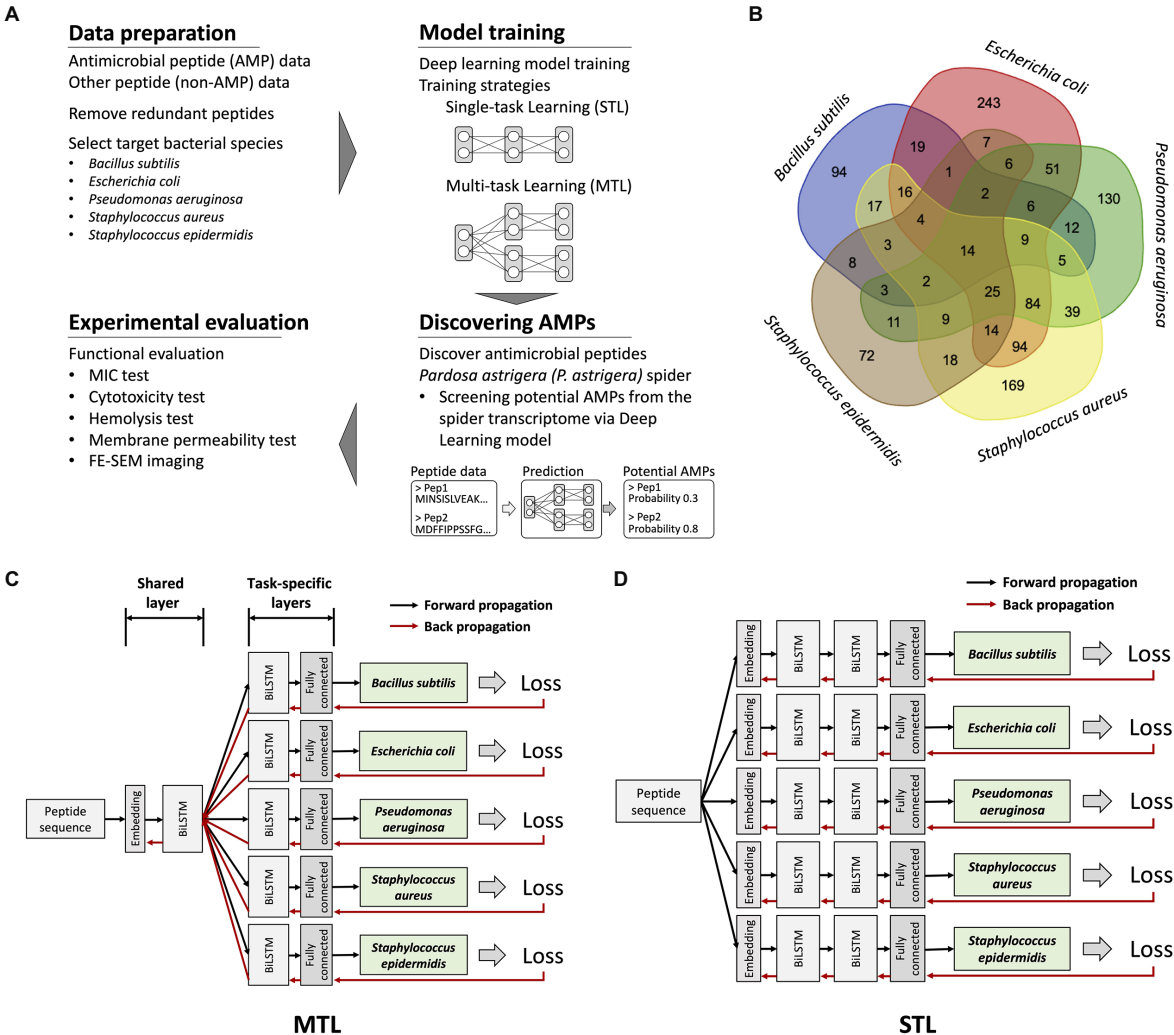
An overview of research workflow, dataset information, and deep learning model structures. **(A)** A schematic representation of discovering novel AMPs from *Pardosa astrigera* spider venom *via* deep learning model. **(B)** A Venn diagram showing the number of antimicrobial peptides targeting each bacterial species. Only 14 peptides exhibited antimicrobial activities for five target bacterial species under a MIC value of 267.7 μg/ml. **(C)** An illustration of the model structure for the multi-task learning (MTL). The model was organized by a single shared layer composed of embedding and BiLSTM layers and task-specific layers for each target composed of BiLSTM and fully connected layers. The loss value of each prediction target was calculated, resulting in five values, which were backpropagated at the same time. **(D)** An illustration of the model structure for the STL. The model was constructed using embedding, BiLSTM, and fully connected layers without parameter sharing through a shared layer.

In this study, an accurate deep learning model for predicting antimicrobial activities against five bacterial strains was developed *via* multi-task learning (MTL), a method that can overcome the lack of data by improving generalization (Wang et al., 2017; Figure 1A). We curated the dataset with AMP and non-AMP data for model training and testing. Due to the extreme data limitation when training the model, prediction targets were restricted to five bacterial species, *Bacillus subtilis* (*B. subtilis*), *Escherichia coli* (*E. coli*), *Pseudomonas aeruginosa* (*P. aeruginosa*), *Staphylococcus aureus* (*S. aureus*), and *Staphylococcus epidermidis* (*S. epidermidis*). The MTL was applied for model training and was compared with the single-task learning (STL) application. The implementation of the MTL resulted in the best-performing model, which was used for screening potential AMPs from the transcriptome of the spider *Pardosa astrigera* (*P. astrigera*). Two spider-derived peptides, PA-Full and PA-Win, were selected and were verified of their antibacterial activities by functional evaluation. Notably, PA-Win was shown to be a strong AMP with low cytotoxicity in mammalian cells. Our results demonstrated that the deep learning model trained by the MTL achieved an improved prediction performance, overcame the data deficiency, and predicted novel AMPs with high cytocompatibility compared with the application of the STL.

## Materials and methods

### Dataset preparation

The AMP data for model training were collected from the database of antimicrobial activity and structure of peptides (DBAASP; Pirtskhalava et al., 2016). The dataset contains 14,708 monomer sequences of peptides with known minimum inhibitory concentration (MIC) values against diverse microbial species. The MIC values in the dataset expressed as micromolar (μM) were converted to microgram per milliliter (μg/ml) for consistency. For peptides that had multiple MIC test results in an identical species, the average value of MIC was calculated and used. Among the AMP data, several peptides had high MIC values that indicated extremely low antibacterial activity. Thus, we filtered the AMPs by a cutoff value of 267.7 μg/ml, which is the lower 95% range of MIC values from the entire AMP data. We obtained the non-AMP data from UniProt using the keyword “NOT antimicrobial” (Bateman et al., 2019).

There were many peptide sequences showing high similarity with other peptides and the peptide sequence redundancy can interfere with model training and performance evaluation. Therefore, the redundancy between training and test datasets as well as within the sequences in the test dataset were controlled by the CD-HIT program with a cutoff value of sequence identity threshold < 0.5 to evaluate the model performance accurately (Fu et al., 2012). For the training data, the cutoff value of 0.9 was used to increase the data for model training. We selected five target bacterial species for prediction based on the amount of the AMP sequences that was greater than 190, of which the positive data for respective species were divided into train and test datasets. The non-AMP data possessed more diverse functionality than the AMP data, so we prepared two types of datasets with ratios of 1:1 and 1:3 between the non-AMP and AMP data, and test datasets were organized with the ratio 1:10 (Table 1).

**Table 1:** The number of peptide sequences used for model training and testing.

Target species	Training dataset (1:1)		Training dataset (1:3)		Test dataset	
	AMP	Non-AMP	AMP	Non-AMP	AMP	Non-AMP
*Bacillus subtilis*	155	155	155	465	60	600
*Escherichia coli*	435	435	435	1,305	160	1,600
*Pseudomonas aeruginosa*	301	301	301	903	107	1,070
*Staphylococcus aureus*	380	380	380	1,140	142	1,420
*Staphylococcus epidermidis*	145	145	145	435	54	540

### Prediction model structure and training

There are various types of deep learning networks, among which a recurrent neural network (RNN)-based model is known as an effective way to process sequential data. Peptide data can be considered sequential data because the function of a peptide is determined by a sequential chain of AA residues. Thus, we organized the model structure based on long-term short memory (LSTM), a type of RNN that can tackle the vanishing or explosion gradient problem of RNN (Hochreiter and Schmidhuber, 1997). In addition, bidirectional long short-term memory (BiLSTM) was implemented to reflect both the forward and backward direction of the peptide in the model prediction.

For model training, the MTL and STL were applied, respectively. In the MTL, a shared layer was connected to five different task-specific layers to predict the antimicrobial activity for each target bacterial species. The model architecture of the STL was identical to that of MTL but without a sharing layer with other prediction targets. The configuration of the model architectures was organized with varying BiLSTM node sizes (Supplementary Table 1). We applied hyperparameters for model training with the Adam optimizer with a learning rate of 1E-4 and a maximum training epoch of 500 (Kingma and Ba, 2014).

The performances of the trained models were measured by precision, recall, *F*1 score, and Matthews correlation coefficient (MCC) as follows:


$$\mathrm{Precision}=\frac{\mathrm{TP}}{\mathrm{TP}+\mathrm{FP}}$$

$$\mathrm{Recall}=\frac{\mathrm{TP}}{\mathrm{TP}+\mathrm{FN}}$$

$$F1\;\mathrm{score}=2\frac{\mathrm{Precision}\times \mathrm{Recall}}{\mathrm{Precision}+\mathrm{Recall}}$$

$$\mathrm{MCC}=\frac{\mathrm{TP}\times \mathrm{TN}-\mathrm{FP}\times \mathrm{FN}}{\sqrt{\left(\mathrm{TP}+\mathrm{FP}\right)\left(\mathrm{TP}+\mathrm{FN}\right)\left(\mathrm{TN}+\mathrm{FP}\right)\left(\mathrm{TN}+\mathrm{FN}\right)}}$$

where TP stands for a true positive number, TN for a true negative number, FP for a false positive number, and FN for a false negative number. We selected the best-performing model in the MTL and STL, respectively, according to the highest *F*1 score.

### Discovering novel AMPs from *Pardosa astrigera* spider transcriptome data

The transcriptome of the venom gland of *P. astrigera* spider obtained from the previous study was utilized (Shin et al., 2020). The unigenes were processed for abundance estimation and open reading frame (ORF) region prediction by RSEM and TransDecoder program, respectively (Li and Dewey, 2011). The predicted coding regions was translated into AA residues, and the translated coding regions were used for discovering novel AMPs by deep learning model.

The translated sequences were prepared for two types of input data, using full sequence under length of 50 AAs and partial sequence of the full sequence by sliding window. The sliding window truncated the sequence for desired length and step size from the original sequence, where the truncates were generated with 20-mer length with a step size of five AA in this study. Two types of prepared input data were fed into the selected best-performing model *via* MTL, and the probabilities of antimicrobial activity against five strains were provided. The two peptides, PA-Full and PA-Win, were selected from the model, and net charge and water solubility were calculated using PepCalc.^1^

### Bacterial strains and cell lines

Bacterial strains of *E. coli* (KCCM 11234), *P. aeruginosa* (ATCC 9027), *B. subtilis* (ATCC 6051), *S*. *epidermis* (ATCC 12228), *S. aureus* (KCCM 11335), and methicillin-resistant *S. aureus* (MRSA, ATCC 33591) were used in this study. Every strain was maintained in Mueller-Hinton broth (MHB, Difco Laboratories, Detroit, MI, United States) under shaking conditions at 37°C. In addition, human cell lines of A549 (ATCC CCL-185), MCF7 (ATCC HTB-22), HaCaT (CLS 300493), NHA (Sciencell 1800), and NHDF (ATCC PCS-201–012) were used. The cells were cultured under a humidified atmosphere with 5% CO2 at 37°C. A549 cells were cultured in RPMI-1640 (Gibco, Grand Island, NY, United States) supplemented with 10% fetal bovine serum (FBS, Gibco) and 1% penicillin and streptomycin (PS, Gibco). HaCaT and NHDF cells were cultured in Dulbecco’s modified Eagle medium supplemented with 10% FBS and 1% PS. MCF7 cells were cultured in minimum essential media (Gibco) supplemented with 10% FBS, 1% PS, and 1 × non-essential amino acids solution (Gibco). Finally, NHA cells were cultured in astrocyte medium (Gibco).

### Antibacterial activity assessment

The MIC values of peptides were measured by performing the broth microdilution method. Bacterial strains were cultured in MHB at 37°C to reach an exponential phase. The bacterial suspension was prepared into 2 × 10^5^ CFU/ml and transferred 50 μl to 96-well microplates. An equal volume of peptides was added to each well, reaching a final concentration from 0.125 to 256 μg/ml of the peptides. The wells containing either only media or the mixture of inoculant and media served as blank and control, respectively. The bacterial growth was measured using a microplate reader (Molecular Devices, Sunnyvale, CA, United States) at 600 nm after incubation at 37°C for 18 h. MIC values of the peptides were determined of the lowest concentration without observable growth of bacteria. To assess the bactericidal effect of the peptides, 50 μl of the samples from each well below the MIC values were plated onto tryptic soy agar (Difco Laboratories) plates. The agar plates were incubated at 37°C overnight, and the minimum bactericidal concentration (MBC) values were defined as the lowest peptide concentrations without any colony formation.

### Measuring bacterial membrane permeabilization

The measurement of outer membrane permeabilization and cytoplasmic membrane depolarization was performed using 1-*N*-phenylnapthylamine (NPN) and 3,3′-dipropylthiadicarbocyanine iodide (DiSC_3_(5)), respectively, as previously described (Shin et al., 2022).

### Field emission scanning electron microscope (FE-SEM) imaging

Morphological changes upon treating peptides were observed using FE-SEM imaging. Target bacteria strains in the exponential phase were diluted in medium to reach 1 × 10^7^ CFU/ml in MHB and seeded onto cover glass coated with poly-L-lysine. After 2 h at room temperature (RT), the bacteria were washed with PBS and treated with 1 × MIC of each peptide for 4 h at RT. The samples were washed with PBS and fixed with 2.5% glutaraldehyde solution at 4°C overnight. After washing with distilled water, bacterial samples were dehydrated with a series of gradient ethanol concentrations (30, 50, 60, 70, 90%, twice for 100%) and then air-dried. Following the platinum coating, bacterial morphology was observed under the FE-SEM sigma instrument (Carl Zeiss Microscopy, Jena, Germany).

### Cytotoxicity assay

For cytotoxicity assay of the peptides, the cells were seeded at a density of 1 × 104 cells/well in 96-well microplates. Various concentrations of peptides ranging from 1 to 256 μg/ml were treated and then incubated for 24 h. The microplate was incubated for 1 h after adding Quanti-Max WST-8 Cell Viability assay solution (Biomax, Seoul, South Korea) to each well. For calculating relative cell viability, the absorbance (Ab) was read using a microplate reader (Molecular Devices) at 450 nm.

### Hemolysis assay

To determine the hemolytic effects, bovine red blood cells (RBCs, Innovative Research, Novi, MI, United States) were used. RBC suspensions of 100 μl were transferred to each microtube and were combined with an equal volume of peptide solution. PBS and 0.1% Triton X-100 were used as the negative and positive controls, respectively. The samples were incubated for 1 h at 37°C and then centrifuged at 3,000 × *g* for 10 min at 4°C. After transferring the supernatants to a 96-well microplate, the Ab was measured using a microplate reader (Molecular Devices) at 450 nm. The hemolytic activity was calculated using the following equation:


$$\mathrm{Hemolytic activity}\;\left(\%\right)=\frac{{\mathrm{Ab}}_{\mathrm{peptide}}-{\mathrm{Ab}}_{\mathrm{PBS}}}{{\mathrm{Ab}}_{\mathrm{Triton}\;X-100}-{\mathrm{Ab}}_{\mathrm{PBS}}}\times 100.$$

### Statistical analysis

All experimental work was independently conducted, and the results were expressed as mean ± standard error of the mean (SEM). The statistical significance of the data was evaluated by performing a one-way analysis of variance (ANOVA) test, followed by Bonferroni correction using GraphPad Prism 9.0 (GraphPad Software, La Jolla, CA, United States). *p*-Values of < 0.05 were considered statistically significant.

## Results

### Training deep learning model for antimicrobial activity prediction *via* MTL

To predict antimicrobial activities targeting a selected set of bacteria, a model should provide several independent prediction results for individual targets. The prediction targets were five different bacterial species, which could be considered multiple related tasks. Multi-label classification could be one approach to model training (Zhang and Zhou, 2013). However, because only 14 peptide sequences were fully labeled for all prediction targets, multi-label classification was not applicable (Figure 1B). Thus, we applied the MTL on non-overlapping datasets by optimizing the model with multiple loss functions (Zhang and Yang, 2017). The model structure was organized as one shared layer connected to several task-specific output layers, otherwise known as hard parameter sharing, and the method effectively prevented an overfitting problem (Figure 1C; Strezoski et al., 2019). During model training, the gradients from the individual prediction target flowed through each task-specific layer and were merged into a shared layer. In comparison, the model for the STL was organized as serially stacked BiLSTM layers without a shared layer, where one model predicts for each target species only (Figure 1D). Considering that the antimicrobial activity of the peptide is exhibited regardless of sequence direction, the BiLSTM-based prediction model was used. Essentially, the STL and the MTL adopted an identical model structure for predicting individual target species, but only the MTL with the shared layer is trained by broad features of AMPs from the integrated dataset. To determine the model structure with better performance than others, we applied various BiLSTM unit sizes and hyperparameters. The input sequence length fed into the model was restricted to be under 50 AAs, as most AMPs in the database rarely exceed 50 AAs. As multiple trained weights were generated during model training, the optimized model in each training strategy and hyperparameter that resulted in the highest *F*1 score on the validation dataset was selected for further evaluation of prediction performance.

**Figure 2:**
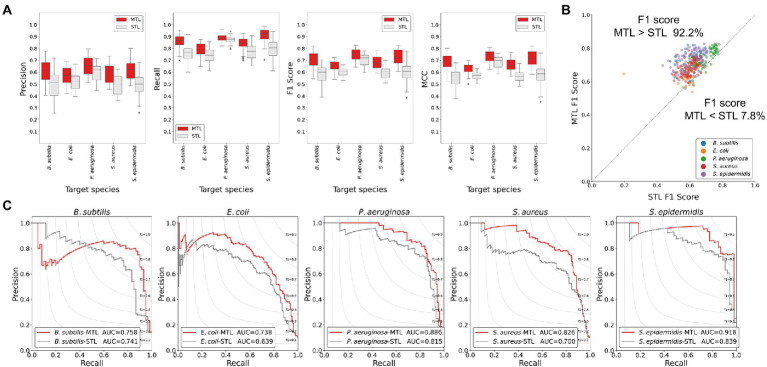
Comparisons of the model performance of the MTL and the STL using the test dataset. **(A)** Boxplots of performance metric results. The MTL scored higher than the STL in precision, recall, *F*1 score, and MCC. **(B)** Scatter plot showing *F*1 scores of the MTL and the STL of the identical model structure. The gray line indicates the equivalent performance of the MTL and the STL in *F*1 score, where dots above the line show that the *F*1 score of the MTL was higher than that of the STL. More than 90% of the dots were above the gray line, suggesting the MTL improved prediction performance effectively. **(C)** Precision-recall curves of the best-performing models in the MTL and the STL. The MTL achieved improved prediction performances of the AUC values.

To evaluate the trained model performance, the prediction performances of the MTL and the STL were compared by performance metrics. As the number of positive class dataset was extremely small for model performance test, the non-AMP dataset was sampled 10 times larger than the positive data for the test dataset for the better evaluation of the model. Hence, three performance metrics, precision, recall, and *F*1 score, were determined. When comparing the box plots of model performance grouped by the STL and the MTL, the MTL showed the best prediction performance in every performance metric and for every target species (Figure 2A). From the scatter plot comparing the prediction performance values trained by the STL versus the MTL under identical model structures, 92.2% of the total results showed improved performances by the MTL than the STL based on the *F*1 score (Figure 2B). Finally, we achieved the best performing model in the STL and the MTL strategies based on the *F*1 score of the test dataset, and the precision-recall curves of the models were exhibited (Figure 2C). A model with the best performance in the MTL outperformed that in the STL for every prediction target species, except for *S. epidermidis* (Table 2). The MTL achieved a precision-recall area under the curve (PRAUC) score of 0.758 for *B. subtilis*, 0.738 for *E. coli*, 0.886 for *P. aeruginosa*, 0.826 for *S. aureus*, and 0.918 for *S. epidermidis*. In addition, the size differences between the non-AMP and the AMP datasets affected the model performance, where the ratio of 1:3 showed better results than that of 1:1 in precision, *F*1 score, and PRAUC, but not recall (Supplementary Figure 1). Overall, the MTL improved the model prediction of the antimicrobial activities of peptides against five species, suggesting that the shared layer boosted the extraction of the latent representation of peptides.

**Table 2:** Prediction results of the best-performing model by multi-task learning (MTL) and single-task learning (STL) on the test dataset.

Bacterial strain	Training strategy	Precision	Recall	PRAUC	*F*1 score	MCC
*Bacillus subtilis*	STL	0.632	0.800	0.741	0.706	0.678
	MTL	**0.750**	**0.900**	**0.758**	**0.818**	**0.802**
*Escherichia coli*	STL	0.587	0.756	0.639	0.661	0.629
	MTL	**0.628**	**0.781**	**0.738**	**0.696**	**0.667**
*Pseudomonas aeruginosa*	STL	0.713	**0.860**	0.815	0.780	0.759
	MTL	**0.789**	0.841	**0.886**	**0.814**	**0.796**
*Staphylococcus aureus*	STL	0.624	0.796	0.700	0.700	0.672
	MTL	**0.736**	**0.845**	**0.826**	**0.787**	**0.766**
*Staphylococcus epidermidis*	STL	**0.681**	0.907	0.839	0.778	0.762
	MTL	0.663	**0.981**	**0.918**	**0.791**	**0.784**

The numbers in boldface indicate the best performance.

### Discovering novel AMPs from the transcriptome of *Pardosa astrigera* spider venom *via* the MTL model

In order to discover novel AMPs from the spider venom, venom gland transcriptome analysis of *P. astrigera*, a wandering spider, was performed (Shin et al., 2020). The venom gland was separated from the spider, and RNA-sequencing and *de novo* assembly were conducted after total RNA isolation (Haas et al., 2013). A total of 149,710 genes with expression levels > 1.0 fragments per kilobase of transcript per million (FPKM) among 169,160 unigenes were identified. The ORF region was predicted by the TransDecoder program, resulting in total 56,373 coding sequence regions (Hwang et al., 2021). The coding regions were translated into AA residues, and the sequences were used for further discovery of AMPs.

**Figure 3:**
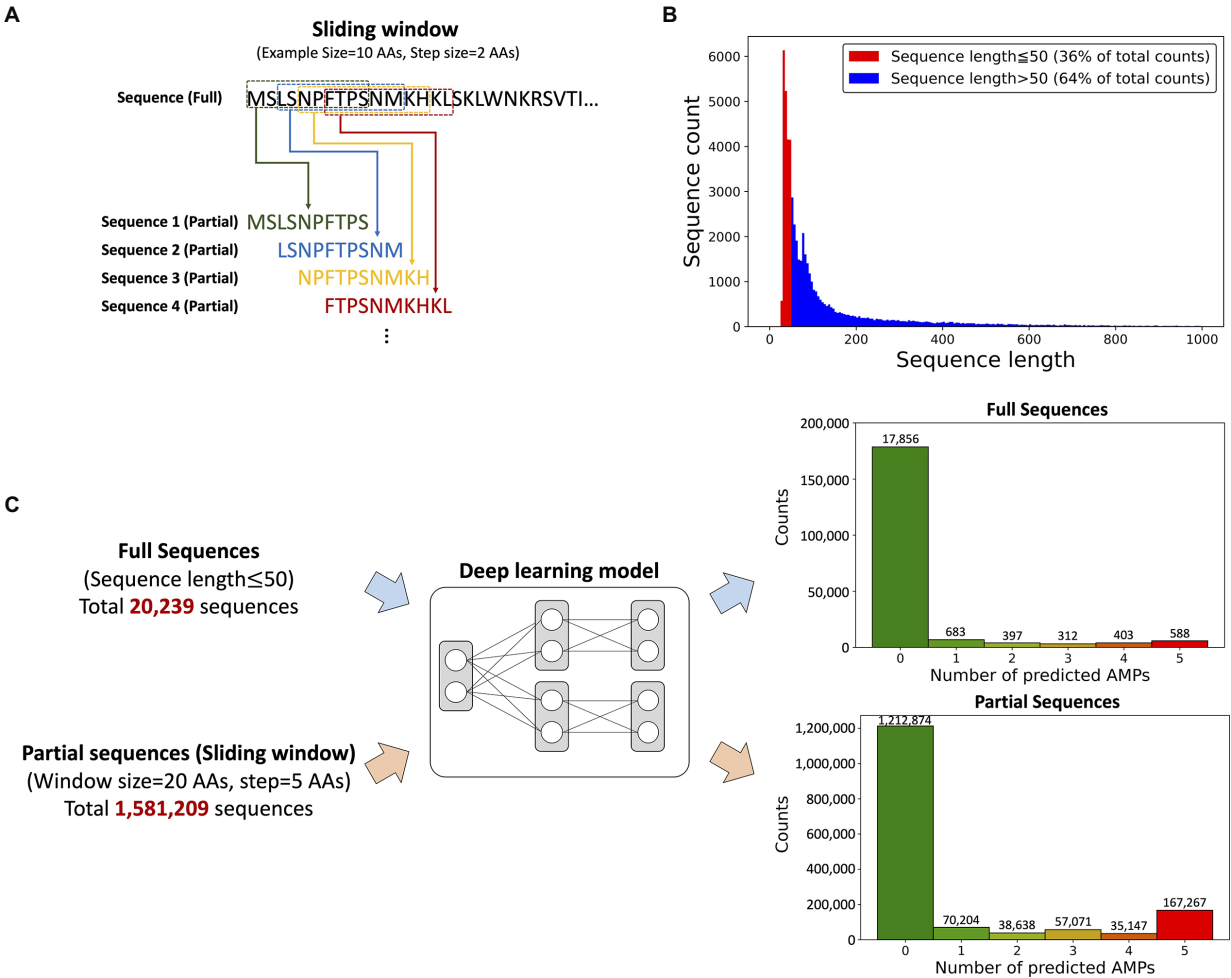
The input data composition and the prediction results from the model. **(A)** A schematic view of the sliding window technique. An example of the truncation of a sequence with a 10-AAs window size and 2-AAs step size. **(B)** Histogram showing AA sequence length of ORF regions from *P. astrigera* transcriptome. The sequences under 50 AAs in length accounted for 36% of the data. **(C)** Sequence prediction results according to the input data type; full and partial sequences. The prediction results from the deep learning model provided the probability of antimicrobial activity for five target bacterial species. The numbers under each histogram represent the counts predicted as AMPs against five bacterial species, where 588 peptides from full sequences and 167,267 peptides from partial sequences of *P. astrigera* were found as AMPs against all the bacterial species.

Transcripts often consist of signal and propeptide sequences that are cleaved to produce the mature protein with a shortened peptide length but full biological activity (Kuhn-Nentwig, 2021). However, the prediction model was designed to have a maximum input sequence length limitation of 50 AAs, leading to a problem that a substantial portion of the peptides with signal and/or propeptide sequences cannot be used for the antimicrobial activity prediction. Thus, we applied the “sliding window” technique for discovering potential AMPs from not only short sequences but long sequences, as 64% of the transcript was over 50 AAs in length (Figure 3A). The “sliding window” technique truncated sequences with the desired length by horizontally moving the “window frame” on an input peptide (Figure 3B). We selected a window size of 20 AAs because the length of most known AMPs is around 20 AAs.

We predicted AMPs from *P. astrigera* data using the input of full sequences under length of 50 AAs and partial sequences by sliding window technique (Figure 3C). The histogram presented the count of peptides by the total number of bacterial targets predicted as AMPs. For example, the case of “2” represents the total sum of AMPs predicted for two species in every combination among five targets. Initially, 20,239 peptide sequences from *P. astrigera* were predicted to have antimicrobial activities without the sliding window technique, and 588 sequences were predicted AMPs against the five bacterial species. When the sliding window technique was implemented using the window size of 20 AAs and step size of 5 AAs, we obtained 1,581,209 partial peptide sequences, of which 167,267 were predicted to be AMPs against five bacterial species. In the case of other window size ranging from 14 to 24, the results are shown in Supplementary Table 2.

Among numerous potential sequences with antibacterial effects, we selected two peptide sequences from each screening method that met the following criteria: FPKM > 10, net charge above + 3, and good water solubility. Each peptide selected from the results with or without the sliding window technique was named PA-Win or PA-Full, respectively (Table 3). The model predicted the two putative AMPs with almost 100% probability, and the peptides were not homologous to any known AMPs found by APD3 and NCBI BLAST (Camacho et al., 2009; Wang et al., 2016). Only uncharacterized proteins with unknown function from diverse spiders were annotated to PA-Win with an *E*-value of 3*E*–13. Notably, the discovery of PA-Win would not have been possible from the prediction model were it not for local activity prediction *via* the sliding window technique. The two selected peptides, PA-Full and PA-Win, were synthesized and used for further experimental evaluation.

**Table 3:** The prediction results and characteristics of the PA-Full and PA-Win.

Name	Sequence	Predicted results of antimicrobial activity (%)					Sequence similarity		Net charge	Water solubility	FPKM
		*Bacillus subtilis*	*Escherichia coli*	*Pseudomonas aeruginosa*	*Staphylococcus aureus*	*Staphylococcus epidermidis*	APD3	BLAST			
PA-Full	WVILSKKIKRKKKEN SDHQTKFSKKVKTKR	100.0	100.0	100.0	99.0	99.8	–	–	+ 10.8	Good	10.8
PA-Win	WKRFHPFRVV RKIFRRRIKR	100.0	100.0	100.0	100.0	100.0	–	Hypothetical protein	+ 9.8	Good	13.5

### Functional evaluation of the PA-Full and PA-Win predicted from the model

The functional properties of the selected peptides were evaluated by *in vitro* experiments. First, PA-Full and PA-Win were tested for their antimicrobial activities against Gram-negative bacteria *E. coli* and *P. aeruginosa*, and Gram-positive bacteria *B. subtilis*, *S. epidermidis*, and *S. aureus*, which are the five strains used for securing the AMP sequence data. As a representative AMP, melittin, a major component of bee venom, was used as a positive control because it rapidly kills microbes (Lee et al., 2013). The bacteria were treated with peptide concentrations between 0.125 and 256 μg/ml; that is, under the cutoff value of 267.7 μg/ml applied for pre-processing the model dataset. As shown in Table 4, the two peptides exhibited significant growth inhibition of the target strains. PA-Win showed stronger antibacterial effects than PA-Full, having MIC values ranging from 1 to 8 μg/ml on every strain. The potency of bacterial eradication was then measured by the MBC of the peptides. The MBC of PA-Win was obtained against every species, whereas PA-Full was measured only on *B. subtilis*. It was confirmed that the two peptides have antimicrobial function against five different strains, and PA-Win was demonstrated to be a strong AMP, showing a strength comparable to melittin.

**Table 4:** The MIC, MBC, and IC50 values of PA-Full and PA-Win.

	MIC (μg/ml)			MBC (μg/ml)			IC_50_ (μg/ml) (95% CI)	
Bacterial strain	PA-Full	PA-Win	Melittin	PA-Full	PA-Win	Cell line	PA-Full	PA-Win
*Bacillus subtilis*	16	2	8	16	2	A549	> 256	65.9 (59.6–72.5)
*Escherichia coli*	256	8	4	> 256	8	HaCaT	> 256	>256
*Pseudomonas aeruginosa*	256	4	16	> 256	8	MCF7	> 256	81.7 (77.6–86.0)
*Staphylococcus aureus*	32	1	2	> 256	4	NHA	> 256	140.3 (133.7–147.4)
*Staphylococcus epidermidis*	256	2	2	> 256	2	NHDF	> 256	178.8 (162.9–196.3)

**Figure 4:**
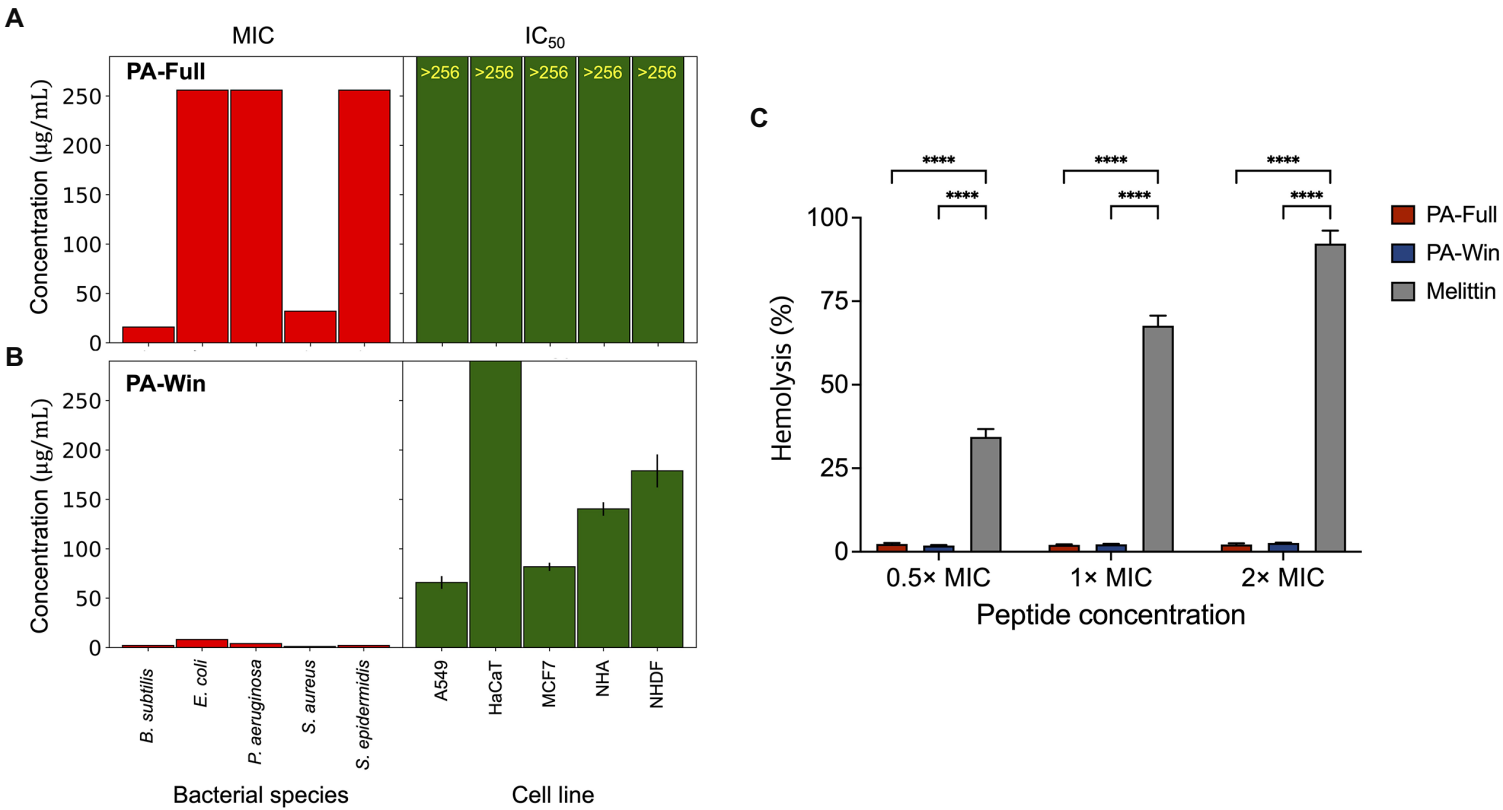
Comparisons between MIC values and cytotoxic effects of PA-Full and PA-Win. **(A,B)** The left bar graph in each panel indicated MIC values of five bacterial species. The right bar graph showed the best-fitted IC_50_ values of five human cell lines with a 95% confidence interval. Both **(A)** PA-Full and **(B)** PA-Win showed MIC values lower than the IC_50_. **(C)** The hemolytic effect of the peptides was compared with that of melittin. Almost no hemolysis was occurred up to 2 × MIC of PA-Full and PA-Win, which was under 3%. On the other hand, melittin caused hemolysis over 90% at 2 × MIC. Data are represented as mean ± SD; ^****^p < 0.0001, one-way ANOVA with Bonferroni correction as a post-test.

The toxicity of the peptides on mammalian cells was also tested to investigate the cytocompatibility for further biological applications. The cytotoxic effect was measured on various cell lines, such as human lung carcinoma (A549), immortalized human keratinocytes (HaCaT), human breast adenocarcinoma (MCF7), normal human astrocytes (NHA), and normal human dermal fibroblasts (NHDF), using the WST assay (Supplementary Figure 2). The half-maximum inhibitory concentration (IC_50_) values and their 95% confidence interval were calculated from three individual tests for each cell line. When treated with PA-Full, cell lines were not affected and remained viable even at peptide concentrations above 256 μg/ml (Figure 4A). The IC50 values of PA-Win on cell lines were at least eight times higher than the highest MIC value, 8 μg/ml (Figure 4B). The hemolytic activities of the peptides were evaluated using bovine erythrocytes. Hemolytic activity was presented as the relative value to 0.1% Triton-X treatment, exhibiting 100% hemolysis, and the concentrations of 0.5 ×, 1 ×, and 2 × MIC of PA-Full, PA-Win, and melittin were tested. When treated with 2 × MIC, PA-Full and PA-Win caused almost no hemolysis which was below 3%, whereas the melittin caused hemolysis over 90% (Figure 4C). Both peptides showed inhibitory concentrations that can exert sufficient antibacterial activity without affecting mammalian cells, and PA-Win was observed to be the stronger AMP among the two peptides.

### Effects of PA-Full and PA-Win on bacterial membrane integrity

**Figure 5:**
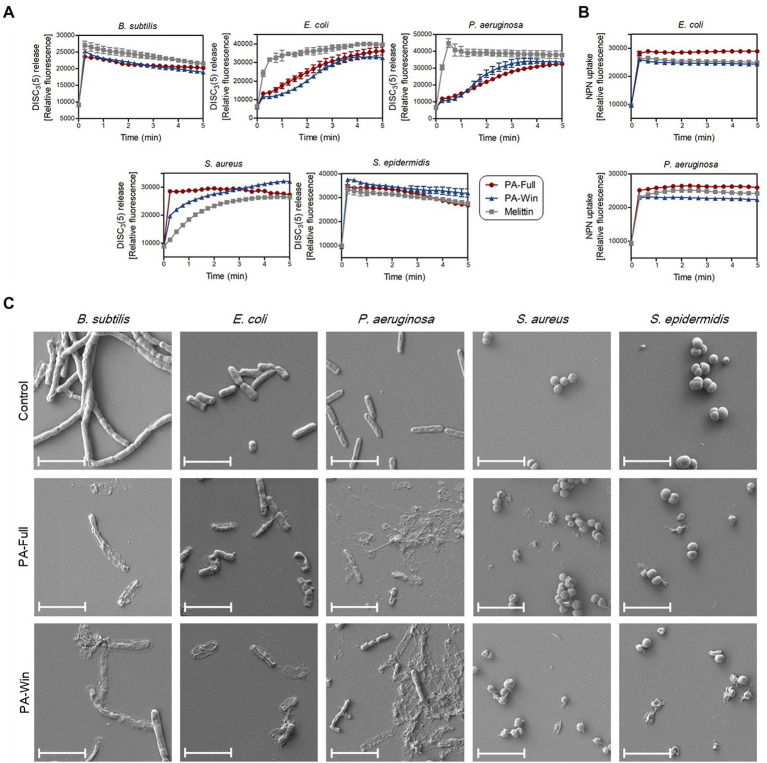
Disruption of bacteria membrane upon PA-Full and PA-Win treatment. **(A)** The cell membrane permeability of DiSC_3_(5) was measured to evaluate the integrity of bacterial cytoplasmic membranes. The increase in fluorescence intensity was observed when Gram-positive and Gram-negative bacteria were treated with 1 × MIC of the peptides, PA-Full, PA-Win, or melittin. **(B)** The permeabilization of the outer membranes of Gram-negative bacteria was observed by the NPN uptake assay. Treatment with PA-Full or PA-Win showed sharp increases in fluorescence intensity comparable to that of melittin. **(C)** Morphologies of bacteria imaged by FE-SEM. Every strain treated with 1 × MIC of peptides for 4 h showed damage to bacterial membranes with blebs and ruptures compared with the negative control treated with an equal volume of PBS. The scale bars represent 4 μm.

It is well known that AMPs exert their antibacterial activity by disrupting the membrane, causing pore formation and leakage of cellular components. Fluorescent dyes DiSC_3_(5) and NPN were used to evaluate the effects of the selected AMPs on bacterial membrane integrity, individually targeting the cytoplasmic and outer membrane. Along with the two selected peptides, 1 × MIC of melittin (shown in Table 3) was again used as a positive control because it kills microbes by membrane disruption. As depicted in Figure 5A, bacterial cells treated with the two respective peptides showed either an instant or time course increase in relative fluorescence intensity by DiSC_3_(5) due to a depolarized cytoplasmic membrane. PA-Full and PA-Win caused sharp increases in the relative fluorescence of Gram-positive strains than Gram-negative bacteria. In Gram-negative strains, the NPN uptake assay was additionally conducted to measure the outer membrane disruption caused by treating with the peptides (Figure 5B). The maximum relative fluorescence intensity was reached within a minute for both peptides, where the relative fluorescence intensity obtained by treating with PA-Full exceeded that by melittin. The results showed that PA-Full and PA-Win effectively led to biomembrane defects.

In agreement with the DiSC_3_(5) and NPN fluorescent measurements, FE-SEM revealed the morphological changes upon peptide treatment (Figure 5C). The control groups were treated with an equal volume of PBS. While the control group retained their normal shape with an intact surface, the bacteria treated with the two respective peptides for 4 h showed apparent damages on cells. The peptide-treated groups showed blebs and deformations on the cell surface and even complete rupture. Altogether, these results indicated that PA-Full and PA-Win permeabilized the cells and disrupted bacterial membranes, like other known AMPs.

## Discussion

Antimicrobial peptides (AMPs) have recently received major attention for the potential new therapeutic agents because of their ability to efficiently kill a broad range of microorganisms by permeating biomembranes, damaging cellular components, and inhibiting metabolic processes (Mwangi et al., 2019). Animal venoms comprise various biological components that are used in predation and defense, such as AMPs. However, identifying potential AMPs from animal venoms *via* conventional experimental methods is time-consuming and labor-intensive. Therefore, we developed a deep learning model that predicts potential candidate AMPs based only on AA sequences and then used the transcriptome data obtained from the venom gland of spider *P. astrigera* to discover novel AMPs.

Spider venom is a rich source of functional biomolecules as it is well known of its diversity in venom components with various effects, such as antimicrobial, analgesic, antimalarial, and anti-arrhythmic activities. Thus, we obtained venom gland transcriptome data from the spider *P. astrigera* to identify novel AMPs. Many studies are based on homology search when handling the transcriptome data for investigating and characterizing animal venoms. However, because homology search is based on the sequence similarity with already discovered peptides, it has limitations in identifying completely novel functional peptides with no resemblance to known peptide sequences. By contrast, the deep learning approach predicts the antimicrobial functionality based on the peptide sequence per se; that is, the antimicrobial activity is predicted based on the input sequences of AAs without considering any physicochemical properties, only using the latent representation of the AA sequences. Thus, the deep learning model can broaden the search for AMPs that the homology search cannot discover.

In order to discover peptides with antibacterial activity against a broad range of bacterial species, we aimed to develop a deep learning model for predicting antimicrobial functionality against five bacterial species. Among the various criteria employed for categorizing AMPs by several databases, a cutoff value of MIC of 267.7 μg/ml was applied to obtain more data in the means of increasing the prediction target species. Lowering the MIC cutoff may possibly identify stronger AMPs than the current model, however, it should limit the prediction target by diminishing the number of peptides that can be utilized to train the model. Finally, the AMP data used for this study were selected based on available peptides that met the minimum number applicable for the deep learning model training. However, about 60% of the peptides were demonstrated to kill only single bacterial species among five target species (Figure 1B). The majority of the positive data was AMPs with narrow-spectrum antimicrobial activity; although such sequences may be active against species that were not tested yet, it still provides information only on narrow-spectrum until experimentally validated. In this context, a prediction model for AMPs without considering species-specific targets will provide AMP sequences with a narrow-spectrum, targeting only one or two strains. Therefore, we designed a species-specific prediction model that provides information about antimicrobial activity against multiple bacterial species, two Gram-negative and three Gram-positive species, to avoid the discovery of narrow-spectrum AMPs.

The data for AMPs against *B. subtilis*, *E. coli*, *P. aeruginosa*, *S. aureus*, and *S. epidermidis* barely met the minimum amount of data for the deep learning approach, while the other strains were not even close. Thus, we developed a deep learning model *via* the MTL approach to overcome and compensate for these data limitations. The MTL is known to improve predictive performance by training multiple related tasks simultaneously, increasing data efficiency, and reducing overfitting (Crawshaw, 2020). Several efforts were made to increase the classification ability of the model using the data size between AMPs and non-AMPs. When it comes to the feature distribution on peptides, the AMP class can be said to be “local,” whereas the non-AMP is scattered as “global,” comprising diverse peptides without distinctive functions. For the model to improve the discrimination between both classes, the test dataset was constructed with AMPs and non-AMPs with a 1:10 ratio. In addition, two types of model training datasets were configured for each target bacterial species with the ratio between AMP and non-AMP as 1:1 and 1:3 for comparison. The MTL showed better predictive performance in the test dataset compared with STL. The weights of the shared layer were tuned by the gradient flows from five different tasks, which may facilitate the shared layer to learn more general latent representations of the peptides. When comparing the results based on the different amount of non-AMP in the training dataset, using a ratio of 1:3 between AMP and non-AMP data in both MTL and STL showed better performance than a ratio of 1:1 in precision. Meanwhile a ratio 1:1 partially performed better in recall metric, which may be due to the imbalance of the training data. A ratio of 1:3 resulted in improved prediction performance based on both the *F*1 score and PRAUC, which denote the balanced model performance, suggesting the distinguishability between AMP and non-AMP has further improved. Finally, based on the highest *F*1 score, we selected the best-performing model for discovering novel AMPs.

The best-performing model was provided with two input data types from the transcriptome of the spider *P. astrigera*, the full sequences and the partial sequences created by the sliding window, for antimicrobial activity prediction. Input data was about 78 times larger in the case of the partial sequences by sliding window and predicted about 284 times larger potential AMPs compared with the full sequence data. The sliding window technique facilitated the expansion of the input data, as well as the prediction results from the same transcriptome data. We selected PA-Full and PA-Win each from the predicted results using the full and partial sequences as the input data. The transcripts showed no homology with known peptides found by NCBI BLAST, resulting in PA-Full with no match and PA-Win with a hypothetical protein of unknown function (*E*-value of 3*E*–13). Both peptides were identified as AMPs through experimental evaluation, and, particularly, PA-Win showed outstanding antimicrobial activity with low toxicity to mammalian cells. Our deep learning model discovered novel AMPs that otherwise cannot be suggested by the traditional methods, including but not limited to BLAST. The model also demonstrated that PA-Win would not have been identified without implementing the sliding window technique. Finally, PA-Win has shown significant antibacterial activity comparable to that of a representative AMP, melittin, against all the tested strains and exhibited cytocompatibility without hemolytic activity, further raising its value. By using the model that we developed, it enables high-throughput prediction of novel functional peptides with low cost and effort, excavating peptides with high potential and high applicability as AMPs, such as PA-Win.

Our model can predict AMP functionality from any sequence data based on AAs, including the transcriptomes of other venomous species. The sliding window technique even accelerates the production of vast prediction results, which can contribute to the acquisition of AMP candidates for next-generation antibiotics. Conclusively, as our MTL-based deep model has successfully discovered an ideal AMP (i.e., PA-Win), we believe the methodology can be applied to the fields of biology with insufficient data for utilizing specific biological data and exploring multiple functional resources, including but not limited to AMPs.

## Data availability statement

The datasets generated for this study can be found at https://github.com/bzlee-bio/AMPSpeciesSpecific.

## Author contributions

MKS and BL conceptualized the study, developed the deep learning model, performed experimental validation, and wrote the manuscript. JSY acquired the funding, coordinated the project, and provided revision for the study. J-SS and WJ supervised the research and edited the manuscript. All authors contributed to the article and approved the submitted version.

## Funding

This work was supported by a grant from the National Institute of Biological Resources (NIBR), funded by the Ministry of Environment (MOE) of the Republic of Korea (NIBR202231204).

## Conflict of interest

The authors declare that the research was conducted in the absence of any commercial or financial relationships that could be construed as a potential conflict of interest.

## Publisher’s note

All claims expressed in this article are solely those of the authors and do not necessarily represent those of their affiliated organizations, or those of the publisher, the editors and the reviewers. Any product that may be evaluated in this article, or claim that may be made by its manufacturer, is not guaranteed or endorsed by the publisher.

## Footnotes

^1^ http://pepcalc.com
