# Supplementary material for: Identifying novel antimicrobial peptides from venom gland of spider Pardosa astrigera by deep multi-task learning
Source: Front Microbiol. 2022 Aug 24;13:971503. doi: 10.3389/fmicb.2022.971503 (PMC9449525; doi:10.3389/fmicb.2022.971503)
Supplement: Supplementary file 1 [file Data_Sheet_1.docx]

Supplementary Material

**Supplementary Table 1**. Information on model structures.

| Model number | MTL | | STL | Model number | MTL | | STL |
| --- | --- | --- | --- | --- | --- | --- | --- |
|  | Shared | Task-specific | BiLSTM  unit size |  | Shared | Task-specific | BiLSTM  unit size |
|  | BiLSTM  unit size | BiLSTM  unit size |  |  | BiLSTM  unit size | BiLSTM  unit size |  |
| 1 | 16 | 16 | 16–16 | 37 | 32–32 | 16 | 32–32–16 |
| 2 | 16 | 32 | 16–32 | 38 | 32–32 | 32 | 32–32–32 |
| 3 | 16 | 64 | 16–64 | 39 | 32–32 | 64 | 32–32–64 |
| 4 | 16 | 16–16 | 16–16–16 | 40 | 32–32 | 16–16 | 32–32–16–16 |
| 5 | 16 | 32–32 | 16–32–32 | 41 | 32–32 | 32–32 | 32–32–32–32 |
| 6 | 16 | 64–64 | 16–64–64 | 42 | 32–32 | 64–64 | 32–32–64–64 |
| 7 | 32 | 16 | 32–16 | 43 | 32–32 | 16 | 32–32–16 |
| 8 | 32 | 32 | 32–32 | 44 | 32–32 | 32 | 32–32–32 |
| 9 | 32 | 64 | 32–64 | 45 | 32–32 | 64 | 32–32–64 |
| 10 | 32 | 16–16 | 32–16–16 | 46 | 32–32 | 16–16 | 32–32–16–16 |
| 11 | 32 | 32–32 | 32–32–32 | 47 | 32–32 | 32–32 | 32–32–32–32 |
| 12 | 32 | 64–64 | 32–64–64 | 48 | 32–32 | 64–64 | 32–32–64–64 |
| 13 | 64 | 16 | 32–16 | 49 | 32–32 | 16 | 32–32–16 |
| 14 | 64 | 32 | 32–32 | 50 | 32–32 | 32 | 32–32–32 |
| 15 | 64 | 64 | 32–64 | 51 | 32–32 | 64 | 32–32–64 |
| 16 | 64 | 16–16 | 32–16–16 | 52 | 32–32 | 16–16 | 32–32–16–16 |
| 17 | 64 | 32–32 | 32–32–32 | 53 | 32–32 | 32–32 | 32–32–32–32 |
| 18 | 64 | 64–64 | 32–64–64 | 54 | 32–32 | 64–64 | 32–32–64–64 |
| 19 | 16–16 | 16 | 16–16–16 | 55 | 64–64 | 16 | 64–64–16 |
| 20 | 16–16 | 32 | 16–16–32 | 56 | 64–64 | 32 | 64–64–32 |
| 21 | 16–16 | 64 | 16–16–64 | 57 | 64–64 | 64 | 64–64–64 |
| 22 | 16–16 | 16–16 | 16–16–16–16 | 58 | 64–64 | 16–16 | 64–64–16–16 |
| 23 | 16–16 | 32–32 | 16–16–32–32 | 59 | 64–64 | 32–32 | 64–64–32–32 |
| 24 | 16–16 | 64–64 | 16–16–64–64 | 60 | 64–64 | 64–64 | 64–64–64–64 |
| 25 | 16–16 | 16 | 16–16–16 | 61 | 64–64 | 16 | 64–64–16 |
| 26 | 16–16 | 32 | 16–16–32 | 62 | 64–64 | 32 | 64–64–32 |
| 27 | 16–16 | 64 | 16–16–64 | 63 | 64–64 | 64 | 64–64–64 |
| 28 | 16–16 | 16–16 | 16–16–16–16 | 64 | 64–64 | 16–16 | 64–64–16–16 |
| 29 | 16–16 | 32–32 | 16–16–32–32 | 65 | 64–64 | 32–32 | 64–64–32–32 |
| 30 | 16–16 | 64–64 | 16–16–64–64 | 66 | 64–64 | 64–64 | 64–64–64–64 |
| 31 | 16–16 | 16 | 16–16–16 | 67 | 64–64 | 16 | 64–64–16 |
| 32 | 16–16 | 32 | 16–16–32 | 68 | 64–64 | 32 | 64–64–32 |
| 33 | 16–16 | 64 | 16–16–64 | 69 | 64–64 | 64 | 64–64–64 |
| 34 | 16–16 | 16–16 | 16–16–16–16 | 70 | 64–64 | 16–16 | 64–64–16–16 |
| 35 | 16–16 | 32–32 | 16–16–32–32 | 71 | 64–64 | 32–32 | 64–64–32–32 |
| 36 | 16–16 | 64–64 | 16–16–64–64 | 72 | 64–64 | 64–64 | 64–64–64–64 |

| Window Size | Number of predicted as AMPs against five bacterial species | | | | | |
| --- | --- | --- | --- | --- | --- | --- |
|  | 0 | 1 | 2 | 3 | 4 | 5 |
| 14 | 1,377,098 | 79,687 | 22,471 | 23,148 | 27,902 | 94,098 |
| 15 | 1,339,022 | 69,099 | 26,689 | 34,597 | 41,336 | 106,592 |
| 16 | 1,190,343 | 101,107 | 45,990 | 85,773 | 51,462 | 135,184 |
| 17 | 1,142,608 | 123,820 | 63,711 | 87,716 | 45,696 | 139,012 |
| 18 | 1,081,032 | 120,063 | 86,565 | 97,912 | 50,237 | 159,715 |
| 19 | 1,126,255 | 78,046 | 56,796 | 105,116 | 58,615 | 163,442 |
| 20 | 1,212,874 | 70,204 | 38,638 | 57,071 | 35,147 | 167,267 |
| 21 | 1,251,754 | 59,013 | 33,912 | 31,276 | 35,496 | 162,274 |
| 22 | 1,268,623 | 44,096 | 37,315 | 27,925 | 30,234 | 158,236 |
| 23 | 1,263,556 | 43,175 | 41,736 | 26,477 | 28,719 | 155,727 |
| 24 | 1,190,697 | 78,411 | 56,544 | 34,416 | 35,817 | 156,251 |

**Supplementary Table 2**. Sequence prediction results according to the partial sequences with varying window size

**Supplementary Figure 1.** Comparisons of the model performances in the MTL and the STL using the test dataset. Boxplots of results for performance metrics (A) precision, (B) recall, (C) *F*1 score, (D) MCC, and (E) PRAUC. The dataset ratio of MTL-1 and STL-1 was 1:1 between AMP and non-AMP, and 1:3 for MTL-3 and STL-3.

**Supplementary Figure 2.** The effects of PA-Full and PA-Win treatment on cell viability. The cell lines (A) A549, (B) HaCaT, (C) MCF7, (D) NHA, and (E) NHDF were treated with peptide concentrations ranging from 1 to 256 μg/mL. The values represent mean ± standard deviation from three independent experiments of cell viability test.
